# Effect of Tanshinone IIA on Gut Microbiome in Diabetes-Induced Cognitive Impairment

**DOI:** 10.3389/fphar.2022.890444

**Published:** 2022-07-11

**Authors:** Yanfang Zheng, Xian Zhou, Chenxiang Wang, Jialin Zhang, Dennis Chang, Wenjing Liu, MingXing Zhu, Shuting Zhuang, Hong Shi, Xiaoning Wang, Yong Chen, Zaixing Cheng, Yanxiang Lin, Lihong Nan, Yibin Sun, Li Min, Jin Liu, Jianyu Chen, Jieping Zhang, Mingqing Huang

**Affiliations:** ^1^ Fujian Key Laboratory of Chinese Materia Medica, College of Pharmacy, Fujian University of Traditional Chinese Medicine, Fuzhou, China; ^2^ NICM Health Research Institute, Western Sydney University, Westmead, NSW, Australia; ^3^ College of Integrated Traditional Chinese and Western Medicine, Fu Jian University of Traditional Chinese Medicine, Fu Zhou, China; ^4^ College of Traditional Chinese, Fu Jian University of Traditional Chinese Medicine, Fu Zhou, China

**Keywords:** tanshinone IIA, diabetes-induced cognitive impairment, inflammation, gut microbiome, short-chain fatty acid, lipid metabolism

## Abstract

Diabetes-induced cognitive impairment (DCI) presents a major public health risk among the aging population. Previous clinical attempts on known therapeutic targets for DCI, such as depleted insulin secretion, insulin resistance, and hyperglycaemia have delivered poor patient outcomes. However, recent evidence has demonstrated that the gut microbiome plays an important role in DCI by modulating cognitive function through the gut–brain crosstalk. The bioactive compound tanshinone IIA (TAN) has shown to improve cognitive and memory function in diabetes mellitus models, though the pharmacological actions are not fully understood. This study aims to investigate the effect and underlying mechanism of TAN in attenuating DCI in relation to regulating the gut microbiome. Metagenomic sequencing analyses were performed on a group of control rats, rats with diabetes induced by a high-fat/high-glucose diet (HFD) and streptozotocin (STZ) (model group) and TAN-treated diabetic rats (TAN group). Cognitive and memory function were assessed by the Morris water maze test, histopathological assessment of brain tissues, and immunoblotting of neurological biomarkers. The fasting blood glucose (FBG) level was monitored throughout the experiments. The levels of serum lipopolysaccharide (LPS) and tumor necrosis factor-α (TNF-α) were measured by enzyme-linked immunoassays to reflect the circulatory inflammation level. The morphology of the colon barrier was observed by histopathological staining. Our study confirmed that TAN reduced the FBG level and improved the cognitive and memory function against HFD- and STZ-induced diabetes. TAN protected the endothelial tight junction in the hippocampus and colon, regulated neuronal biomarkers, and lowered the serum levels of LPS and TNF-α. TAN corrected the reduced abundance of *Bacteroidetes* in diabetic rats. At the species level, TAN regulated the abundance of *B. dorei*, *Lachnoclostridium* sp. YL32 and *Clostridiodes difficile*. TAN modulated the lipid metabolism and biosynthesis of fatty acids in related pathways as the main functional components. TAN significantly restored the reduced levels of isobutyric acid and butyric acid. Our results supported the use of TAN as a promising therapeutic agent for DCI, in which the underlying mechanism may be associated with gut microbiome regulation.

## Graphical Abstract

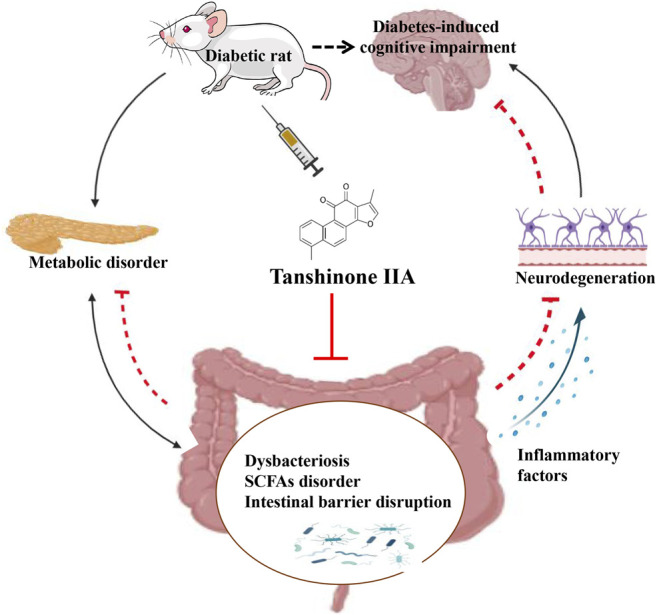

## 1 Introduction

Recent epidemiological and clinical studies have linked both type I and type II diabetes to an increased risk for cognitive dysfunction and memory loss as serious complications of the disease (Salas and De Strooper, 2019). Diabetes-induced cognitive impairment (DCI) is characterised by reduced performance on multiple cognitive domains, particularly in the hippocampus, the temporo-occipital regions, and several frontal areas (Wang et al., 2020). The neurophysiological changes underpinning cognitive dysfunction in type I diabetes remain unclear. Studies of electroencephalograms in adults (Brismar et al., 2002) and adolescents (Hyllienmark et al., 2005) with type I diabetes revealed significant reductions in fast brain wave (α, β, and ɣ) activity, particularly in the temporo-occipital regions (van Duinkerken et al., 2009; McCrimmon et al., 2012). A significant regional variation in cerebral perfusion was also detected in the cerebellum, frontal brain, and frontotemporal brain (Dejgaard et al., 1991; Jiménez-Bonilla et al., 2001; Salem et al., 2002). The use of magnetic encephalography also revealed abnormalities in functional magnetic fields and the neural connectivity of the brain at the scalp of people with type I diabetes (van Duinkerken et al., 2009). Moreover, many studies have supported a link between altered brain structure and clinical parameters in type II diabetic patients (Rosenberg et al., 2019). For instance, brain atrophy (Bruehl et al., 2009), reduced hippocampal volume (Bruehl et al., 2009b; Hayashi et al., 2011), and increased amount of cerebrospinal fluid were all positively correlated with high body mass index, higher level of hemoglobin A1C, and disease duration in type II diabetic patients (Last et al., 2007; Kumar et al., 2008; Brundel et al., 2010; Lee et al., 2013; Yang et al., 2015).

DCI can occur at an early stage of diabetes and has become a major public health risk affecting the aging population (Xu et al., 2017). The cellular and molecular mechanisms underlying DCI were thought to be related to depleted insulin secretion, insulin resistance, hyperglycaemia, systemic inflammation, and neuroinflammation. However, to date, clinical attempts against these therapeutic targets have not shown satisfactory outcomes (Wang et al., 2020).

Increasing evidence has shown that the gut microbiome is linked with the progression of DCI by modulating cognitive function through the gut–brain crosstalk (Xu et al., 2017; Agustí et al., 2018; Bioque et al., 2021). The gut–brain crosstalk, known as the “microbiota–gut–brain axis”, dually controls and regulates the body’s metabolism and neuroendocrine stability (Xu et al., 2017; Cryan et al., 2019). Although the composition of the gut microbiome is complex and varies among individuals, the occurrence of DCI is mainly associated with the major composition of the microbiome, particularly probiotics, *via* endocrine, immune, and neural pathways (Qin et al., 2012; Forslund et al., 2015). For example, Savignac et al. (2015) suggested that the species *Bifidobacterial longum* 1714 improved learning and memory function in BALB/c mice. In addition, the restoration of *Lactobacillus acidophilus*, *L. fermentum* (Davari et al., 2013) and *L. paracasei* HII01 (Chunchai et al., 2018) resulted in reversed deterioration of brain function in diabetic patients through the actions of increased serum insulin level, improved glucose tolerance, and reduced systemic inflammation and neuroinflammation (Cani et al., 2007; Solas et al., 2017; Chunchai et al., 2018; Arnoriaga-Rodríguez and Fernández-Real, 2019). This shows that probiotics and gut microbiome–regulating agents may play a significant role in the management of DCI (Thakur et al., 2019). The underlying mechanism is likely to be associated with the restored diversity and abundance of beneficial bacterial species and their metabolites, such as short-chain fatty acids (SCFAs) (Arnoriaga-Rodríguez and Fernández-Real, 2019).

*Salvia miltiorrhiza* Bunge (SM) is a well-known Chinese medicinal plant used in the treatment of cardiovascular diseases (Ji et al., 2000; Chun-Yan et al., 2015). It has especially attracted great interest for its use in diabetes management, which can be attributed to its ability to reduce oxidative stress and inflammation (two major risk factors of diabetes) (An et al., 2017). Tanshinone IIA (TAN), an active lipophilic component in SM, possess a diverse array of pharmacological properties, including antioxidant, anti-inflammatory, and anti-cancer effects (Ansari et al., 2021). In particular, TAN has been shown to be effective in treating diabetes and neurological disorders (Jia et al., 2019). TAN significantly improved glucose metabolism, reduced insulin resistance, and regulated lipid metabolism in a diabetic rat model *via* the nuclear factor kappa B–mediated inflammatory pathway (Yuan et al., 2018). The TAN treatment also improved hippocampus-dependent memory in diabetic rats by reducing neuronal apoptosis and ameliorating diabetic cardiomyopathy. This was achieved through the antioxidant activity of TAN by suppressing endoplasmic reticulum stress activation (Chen et al., 2018; Tao et al., 2019). Previous findings have demonstrated the therapeutic potential of TAN in ameliorating DCI. However, its mechanism of action in relation to the gut microbiome as an important pathophysiological component of DCI is unclear. The present study aimed to assess the pharmacological action of TAN against DCI and to explore its mechanisms of action for neuroprotective activity *via* gut microbiome regulation.

## 2 Materials and Methods

### 2.1 Animals and Experimental Design

Forty male Sprague-Dawley (SD) rats aged 6 weeks (200 ± 20 g) were purchased from Shanghai Silaike Experiment Animal Co., Ltd. (China). The SD rats were maintained at 25°C with 80% humidity, a 12 h light/dark cycle, and food and water ad libitum for 2 weeks prior to the commencement of the experiment. The SD rats (*n* = 40) were randomly divided into two groups. Seven rats were assigned to the control group; the other 33 were used to induce diabetes. The control group (*n* = 7) were fed a basal diet supplied by the Animal House affiliated with Fujian University of Traditional Chinese Medicine. The other 33 SD rats were supplied with a high-fat/high-glucose diet (HFD) consisting of 10% fat, 15% sucrose, 4% cholesterol, 10% yolk powder, 0.3% cholate, and 60.7% regular chow (Feng et al., 2018; Chen et al., 2021). After 6 weeks, the 33 SD rats were injected with streptozotocin (STZ, IP, 25 mg/kg) twice within a 72 h interval. Five days after the second STZ injection, the fasting blood glucose (FBG) level was tested using blood from the tail vein. Twenty-one SD rats that exhibited an FBG level higher than 11.1 mmol/L were randomly divided into three groups: the model group (*n* = 7), the TAN-treated (TAN) group (*n* = 7), and the metformin (MET) group (*n* = 7). The remaining 12 SD rats, which possessed an FBG level lower than 11.1 mmol/L, were excluded from the study. The HFD-feeding regimen was ceased from week 7; the SD rats in the control and model groups were supplied with 0.9% saline (10 mL/kg/day), and the TAN group was administered TAN (30 mg/kg/day, S31459, Shanghai Yuanye Bio-Technology Co., Ltd., ≥95%, catalogue number H08A9W57777) by intragastric administration for 8 consecutive weeks. The MET group was treated with metformin hydrochloride (100 mg/kg/day, oral gavage, Sino-American Shanghai Squibb Pharmaceutical Co., Ltd.) at the same volume as for the control and model groups for 8 consecutive weeks. At the end of the treatment (week 14), the animals were fasted for 12 h and anesthetised with 20% Ulatan (1 g/kg, IP) before the blood was collected *via* the abdominal aorta. The blood samples were then centrifuged at 500 *g* for 15 min, and the supernatants were collected for analysis. The study was approved by the Ethical Committee of Fujian University of Traditional Chinese Medicines. The *Guide for the Care and Use of Laboratory Animals* was followed in this study (York et al., 2012).

### 2.2 Fasting Blood Glucose Test

For the FBG test, the animals were fasted for 9 h before their tail blood was collected and tested on blood glucose test strips measured by a glucometer (Roche, United States). The FBG was measured prior to the injection of STZ (week 0), after the STZ injection (week 6), and every 2 weeks during the TAN treatment period (weeks 8, 10, 12, and 14). The blood glucose measurements at each time point were conducted three times for each animal, and the averaged values were recorded.

### 2.3 Behavioral Testing

The Morris water maze (MWM) test was used to evaluate the spatial learning and memory function. The training and probe tests were performed on week 14. The animals were fasted 4 h before each experiment to keep them active. The MWM consisted of a circular pool (150 cm diameter × 60 cm height) filled to a depth of 30 cm with water at a temperature of 25 ± 2°C. The maze was divided into four equal quadrants, marked as north, south, east, and west, and the targeted quadrant was set in the centre of the northwest quadrant with a hidden circular platform (6 cm diameter).

During the training phase (day 1–day 4), the rats entered the maze through each of the four quadrants in order, and the movement and averaged escape latency times to the target quadrant were recorded using a tracking system for 4 consecutive days. On day 5, the platform was removed, each rat entered the water maze through the southeast quadrant, and a 90 s probe trial was performed. The escape latency time and platform-crossing times were recorded.

### 2.4 Histopathological Assessment

The histopathological assessment was conducted by hematoxylin and eosin (H&E) staining following a previous study (Liu et al., 2020). Briefly, brain and colon tissues were collected, perfused with 4% paraformaldehyde phosphate buffer, and then dehydrated with ascending concentrations of ethanol (50%, 70%, 80%, 95%, and 100%, for 5 min each). The tissues were subsequently cleared with xylene, embedded in paraffin, and sectioned into 4 μm thickness. Each section was perfused in xylene (for 10 min twice) to remove the wax. The sectioned tissues were soaked in descending concentrations of ethanol (100%, 95%, 90%, 80%, 70%–0%, for 5 min each). The sections were stained with hematoxylin for 5 min, washed with running water for 1 min, soaked in an alcoholic solution of 1% hydrochloric acid for 3 s, rinsed with running water for 10 min, and stained with eosin for 3 min. This was followed by soaking in 95% ethanol (for 5 min twice), 100% ethanol (for 5 min twice), and xylene (for 2 min three times). Finally, the stained sections were sealed, mounted using Permount™ Mounting Medium, and imaged and analysed using a light microscope (Nikon 55i microscope, Nikon, Japan).

### 2.5 Immunoassays

The hippocampal and colon tissues were washed twice with ice-cold phosphate-buffered saline (PBS). The whole-cell protein lysates were extracted with a lysis buffer supplemented with a protease inhibitor mixture (Solarbio, Beijing, China). A bicinchoninic acid protein kit (Shanghai Beyotime, Ltd., Co.) was used to quantify the protein concentration. An equal amount of protein samples from each group were subjected to sodium dodecyl sulfate polyacrylamide gel electrophoresis, and the proteins were transferred to nitrocellulose membranes (Millipore Corporation, Billerica, MA 01821, United States). After blocking with 5% skim milk at room temperature for 1.5 h, the membranes were incubated with the following primary antibodies overnight at 4°C: zonula occludens-1 (ZO-1, 1:1,000, catalogue number: 21773-1-AP), occludin (1:500, catalogue number: 13409-1-AP), and glial fibrillary acidic protein (GFAP, 1:1,000, catalogue number: 23935-1-AP), which were all purchased from Proteintech (United States); phosphorylated tau protein (p-Tau, 1:1,000, catalogue number: ab109390) and tau protein (Tau, 1:1,000, catalogue number: ab254256), purchased from Abcam (United Kingdom). β-actin was used as the internal control (1:3,000, catalogue number: 60008-1-Ig), purchased from Proteintech (United States). Subsequently, the blots were washed in PBS solution with 0.1% Tween™ 20 (Thermo Fisher Scientific, Australia) three times (for 3 min each time) and incubated with anti-mouse/anti-rabbit horseradish peroxidase–conjugated secondary antibody (Proteintech, United States) for 1.5 h. The images of the targeted bands were taken using a ChemiDoc XRS plus imaging system (Bio-Rad, Hercules, CA, United States), and intensity was quantified by ImageJ software. The quantitative data are presented as the ratio of the intensity of the targeted protein to that of β-actin.

### 2.6 Gut Metagenomic Sequencing and Data Analysis

At the end of the treatment on week 14, the fecal sample from each rat was collected in a 1.5 mL centrifuge tube on ice prior to the postmortem. The fecal samples were sent to Wekemo Tech Group Co., Ltd., Shenzhen, China, for the gut metagenomic sequencing and data analysis. DNA was extracted based on the methods described in a previous study (Sun et al., 2019), and the purity and concentration of the DNA were detected by agarose gel electrophoresis. Paired-end metagenomic sequencing was performed on the Illumina NovaSeq platform (United States) with an insert size of 350 bp and paired-end reads of 150 bp for each sample. Low-quality and ambiguous bases from the raw reads were removed (Trimmomatic parameter: illuminaclip: adapters path: 2:30:10, sliding window: 4:20, minlen: 50). The remaining reads that were aligned to the rat’s genome reference and host DNA contamination were removed to obtain the clean data (Bowtie 2 parameter set as ‘very sensitive’). Species diversity and composition were analysed and classified by the Kraken2 (2018) program. Bayesian Reestimation of Abundance with KrakEN (Bracken) was used as the statistical method to compute the abundance in the DNA sequences from each metagenomic sample. The sequence number percent of each sample in total sequences from kingdom to species was obtained based on the results of Bracken. Principal component analysis (PCA) was used to show the overall difference in species composition among the groups. Clustering analysis was used to investigate the similarity of species composition among the samples. Linear discriminant analysis (LDA) effect size (LEfSe) was used to identify the feature species with high abundance in each group. LDA > 2 and LDA > 4 were set as two thresholds to differentiate the species that represented high abundance.

Gene function analysis was performed using the HMP Unified Metabolic Analysis Network (HUMAnN) 2.0 program, which compared the DNA sequences after quality control and host sequence removal with those in the UniProt Reference Clusters 90 (UniRef90) database. The default HUMAnN 2.0 comparison parameters were set as translated_query_corverage_threshold = 90.0, prescreen threshold_0.01, evalue_threshold = 10, and translated_subject_coverage_threshold = 50.0, and the low-quality reads were removed accordingly. The reads per kilobase per million of each protein in UNniRef90 were then used to compare with the clean reads of each sample to obtain the function database, Kyoto Encyclopedia of Genes and Genomes (KEGG), which corresponds to their relative abundance of functions. LEfSe and Dunn’s test were used to identify the feature KEGG pathway, KEGG orthologs (KOs), and KEGG module in each group. The Circos plot was used to visualize the top 10 feature KEGG pathways in each sample.

### 2.7 Determination of Fecal Short-Chain Fatty Acids

Following the collection of fecal samples, the preparation and purification steps were followed according to the previous protocol with a slight modification (Xie et al., 2015). The samples were derivatized and analysed using an ultra-performance liquid chromatography coupled with triple-quadrupole tandem mass spectrometry (UPLC-TQ-MS) system (Acquity UPLC-XEVO TQS, Waters Corp., Milford, MA, United States). QuanMET software (V2.0, Metbo-Profile, Shanghai, China) was used to process the original data files generated by UPLC-MS/MS, and peak integration, calibration, and quantification were performed for each metabolite of SCFAs, including acetic acid, propionic acid, isobutyric acid, butyric acid, 2-methylacetic acid, isovaleric acid, valeric acid, and caproic acid (all purchased from Sigma, China).

### 2.8 Statistical Analysis

Statistical analyses were performed using GraphPad Prism 9.0 software. The results obtained from seven SD rats per group were expressed as averaged values with mean ± standard deviation or standard error of the mean. Two-way repeated measures analysis of variance (ANOVA) was used to show the statistical differences of means in each treatment group at the same time point for the FBG and MWM tests. One-way ANOVA, followed by Tukey’s multiple-comparison test or unpaired *t* test, was used to show the statistical differences between groups. A *p* value <0.05 was considered statistically significant. We also performed the ROUT test in GraphPad Prism 9.0 to exclude outliers.

## 3 Results

### 3.1 TAN Decreased Fasting Blood Glucose in Rats With Diabetes Induced by a High-Fat/High-Glucose Diet and Streptozotocin

**FIGURE 1:**
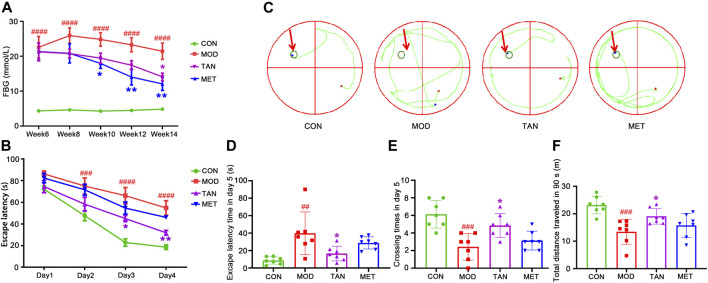
The changes in blood glucose level and cognitive function in response to TAN treatment (*n* = 7 rats per group). **(A)** TAN time-dependently reversed the HFD and STZ-induced high blood glucose level (mmol/L) in diabetic rats from week 6 to week 14. ^####^ *p* < 0.0001 vs*.* CON group at the same time point. *p < 0.05, **p < 0.01 vs*.* MOD group at the same time point as analysed by two-way repeated measures ANOVA analysis. **(B)** TAN treatment exhibited time-dependent effect in reducing escape latency time (s) in the training phase from day 1 to day 4. The escape latency time was compared among the groups at the same time point by two-way repeated measures ANOVA. ^####^ *p* < 0.0001 vs*.* CON group at the same time point. *p < 0.05, **p < 0.01 vs*.* MOD group at the same time point. **(C)** Trajectory chart (green color) represents occupancy across the entire 90 s trial in the probe test (day 5). The green circles on the northwest platform refer to the target platform. **(D)** Statistical analysis by one-way ANOVA showed that TAN treatment significantly increased the time spent in the target quadrant and **(E)** platform-crossing times compared with the MOD group **(F)** TAN significantly restored the total distance travelled in 90 s (m) compared with the MOD group. Results are shown as the mean ± standard deviation. ^*##*^ *p* < 0.01, ^###^ *p* < 0.001 vs*.* CON group and *p < 0.05, **p < 0.01 vs. MOD group. TAN, tanshinone IIA; CON, control; MOD, model.

The FBG test was conducted to monitor the changes in blood glucose level every 2 weeks from week 6 to week 14. As shown in Figure 1A, the FBG level in the model group (22.57 ± 8.19 mmol/L) was significantly higher than that of the control group (4.34 ± 0.21 mmol/L, *p* < 0.0001, *n* = 7 per group) on week 6, whereas no statistical difference in FBG was detected between the model and TAN, and model and MET groups (*p =* 0.9540 and *p* = 0.9619, respectively, *n* = 7 per group). The model group repeatedly exhibited significantly higher FBG levels in comparison with the control group on week 8 (*p* < 0.0001), week 10 (*p* < 0.0001), week 12 (*p* < 0.0001), and week 14 (*p* < 0.0001), suggesting that a 6-week treatment with HFD and STZ injections induced a marked increase in blood glucose level. The TAN treatment showed a time-dependent FBG-reducing effect, with a significantly lowered FBG level from 21.2 ± 6.8 mmol/L on week 6 to 14.1 ± 2.9 mmol/L on week 14 (*p* = 0.0243). MET also significantly reduced FBG on week 10 (17.9 ± 3.8 mmol/L, *p* = 0.0395), week 12 (14.0 ± 6.1 mmol/L, *p* = 0.0024), and week 14 (12.1 ± 4.9 mmol/L, *p* = 0.0021). There was no significant difference in the FBG level between the TAN and MET treatments compared at the same time points throughout the trial (*p* > 0.5482).

### 3.2 TAN Improved the Spatial Learning and Memory Function in Diabetic Rats

As shown in Figures 1A,B, significantly greater escape latency time was detected in the model group from day 2 (75.0 ± 19.7 s) to day 4 (48.9 ± 9.4 s) compared with the control group (day 2: 47.6 ± 12.4 s, *p* = 0.0008; day 4: 16.8 ± 3.1 s, both *p* values <0.0001) at the same time point during the training phase, suggesting impaired cognitive function in the diabetic animals. MET did not show a significant effect in reducing the escape latency time during the training phase (*p* > 0.3481) compared with the model group. Remarkably, the TAN group showed significantly reduced latency time on day 3: 45.0 ± 3.4 s (*p* = 0.0148) and day 4: 31.8 ± 5.7 s (*p* = 0.0067) compared with the model group. In addition, the escape latency time in the TAN group was comparable with that of the MET group from day 1 to day 4 without any significance (*p* > 0.05).

The probe test results (day 5) of the spatial learning capacity test are shown as trajectory charts in Figure 1C. The rats in the control group took the shortest route in finding the targeted quadrant (northwest). In contrast, the rats in the model group presented a weaker spatial learning capacity, as evidenced by a messy trajectory from the starting point to the targeted quadrant. TAN-treated rats, however, displayed fewer movements and a relatively shorter trajectory compared with the model group. It was observed that the animals in the MET group did not show any obvious change in the trajectory when compared with those of the model group.

The statistical comparison of the escape latency time and crossing times on day 5 is shown in Figures 1D,E. For the escape latency time, the model group showed a significantly longer duration (39.8 ± 24.5 s) than the control group (8.6 ± 4.1 s), with *p* = 0.0039. The TAN group significantly shortened the escape latency time (14.1 ± 5.8 s) compared with the model group (39.8 ± 24.5 s, *p* = 0.0346). MET did not show any significant change in the escape latency time compared with the model group (28.7 ± 7.0 s vs*.* 39.8 ± 24.5 s, respectively, *p* = 0.4969). There was no significant difference in the escape latency time between the TAN group and the MET group (*p* = 0.4150).

Similarly, the rats in the model group averaged less platform-crossing times (2.4 ± 1.5) than the control group (6.1 ± 1.6, *p* = 0.0003). The TAN group showed significantly more crossing times than the model group (4.9 ± 1.3 vs*.* 2.4 ± 1.5, respectively, *p* = 0.014). The MET group did not exhibit a significant change compared with the model group (3.1 ± 1.1 vs*.* 2.4 ± 1.5, respectively, *p* = 0.7444). The crossing times in the TAN group were comparable with those of the MET group (4.9 ± 1.3 vs*.* 3.1 ± 1.1, respectively, *p* = 0.1069). In addition, the total distance travelled by the animals was assessed within the 90 s in the probe test (day 5) among the four groups (Figure 1F). The animals in the model group had a significantly shorter distance (13.4 ± 4.5 m) compared with those of the control group (23.2 ± 3.2 m, *p* = 0.0004). The TAN group (19.1 ± 2.8 m) displayed a significantly longer distance than the model group (*p* = 0.0480), whereas the MET group (15.7 ± 4.3 m) did not exhibit a significant difference compared with the model group (*p* = 0.6741). There was no significant difference in the total distance between the TAN and the MET group (*p* = 0.3733).

### 3.3 TAN Alleviated the Pathological Changes in the Hippocampal and Colon Barrier in Diabetic Rats

**FIGURE 2:**
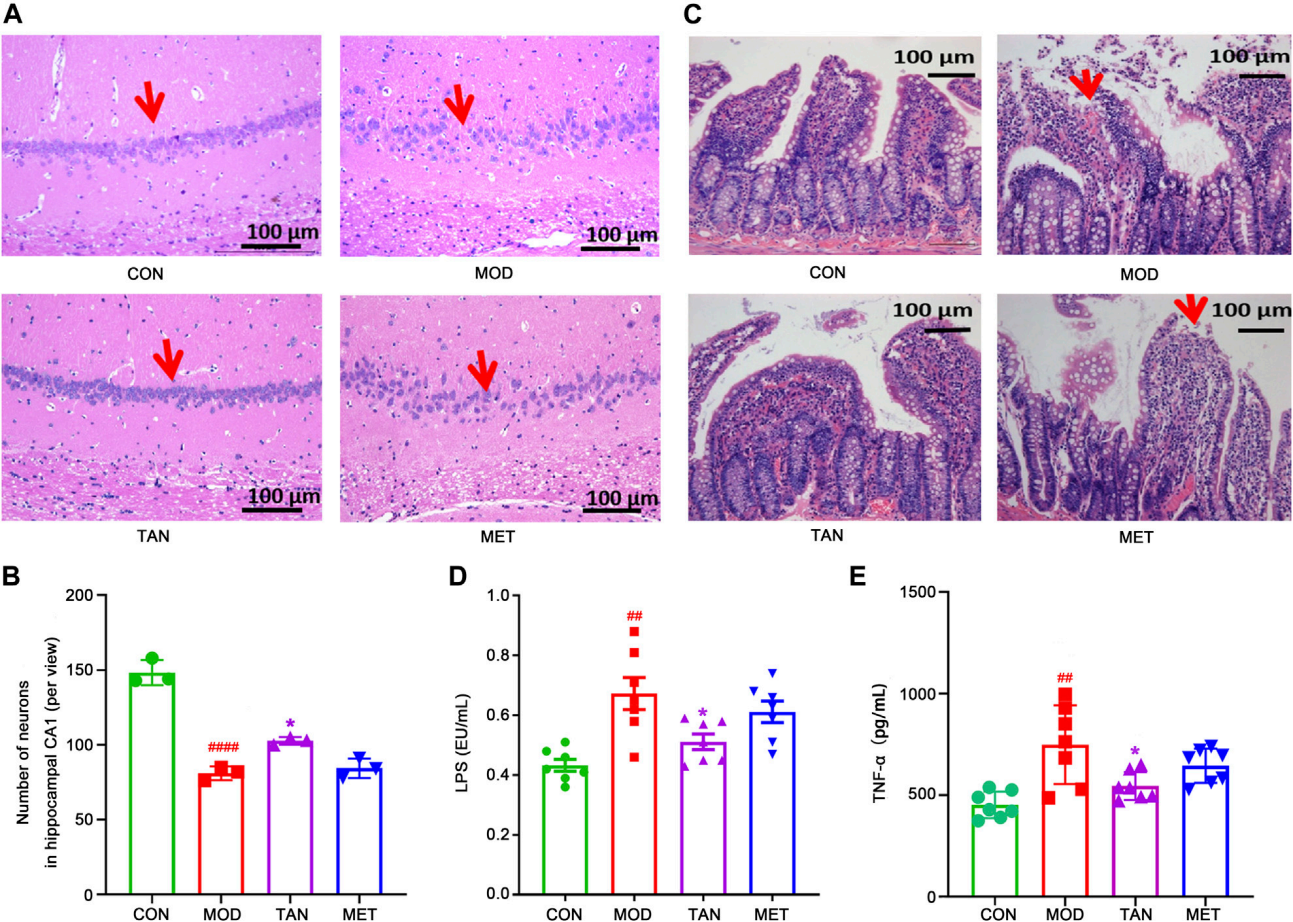
Effect of TAN on histopathologic changes in the rat hippocampal CA1 region and colon tight junction. **(A)** Representative sections of H&E staining (magnification 200×). Red arrow points to the package of neurons. **(B)** Statistical analysis of the number of neurons in the hippocampal CA1 area (per view, *n* = 3). ^####^ *p* < 0.0001 vs*.* CON group and *p < 0.05 vs*.* MOD group by one-way ANOVA. **(C)** Representative sections of H&E staining (magnification 200×) on colon tight-junction area in the CON, MOD, and TAN groups. Red arrow represents the damaged villi. TAN treatment reduced plasma levels of LPS **(D)** and TNF-α **(E)** in the HFD and STZ-induced diabetic rats, *n* = 7 rats in each group. ^#^ *p* < 0.05, ^##^ *p* < 0.01 vs*.* CON group and *p < 0.05 vs*.* MOD group by one-way ANOVA analysis. TAN, tanshinone IIA; CON, control; MOD, model; H&E, hematoxylin and eosin; ANOVA, analysis of variance.

The neuroprotective effect of TAN was assessed by hematoxylin and eosin (H&E) staining. In Figure 2A, it can be seen that the pyramidal neurons were closely and regularly packed with a normal morphology in the control group, whereas fewer and loosely packed pyramidal neuronal cells (red arrows) were identified in the model group, with nuclear fragmentation (karyorrhexis) and cell shrinkage with condensed nuclei (pyknosis). The TAN treatment resulted in an increased number of neurons and closely packed, similar to those in the control group. The MET group did not demonstrate an obvious improvement in the morphology, which remains similar to that of the model group. The statistical analysis (Figure 2B) indicated that the population of neurons in the CA1 area per view in the model group was significantly lower than that of the control group (81.0 ± 4.6 vs*.* 148.3 ± 8.4, respectively, *p* < 0.0001). The TAN group significantly restored the neuron populations when compared with the control group (102.7 ± 2.5 vs*.* 81.0 ± 4.6, respectively, *p* = 0.0308). No significance in the neuron number for the MET treatment was observed (84.3 ± 6.5 vs*.* 81.0 ± 4.6, respectively, *p* = 0.9289). The number of neurons in the TAN group was not significantly different from that of the MET group (*p* = 0.0610).

Colon morphology was evaluated by H&E staining to assess the protective effect of TAN in relation to the gut microbiome in HFD- and STZ-induced DCI. As shown in Figure 2C, the control group exhibited normal morphology of the colon section, with a distinct epithelial barrier and villi (red arrows), and a normal structure of the glandular epithelium. In contrast, the model group revealed serious pathological changes in the colon sections, including impaired epithelial barrier integrity and villi, structural damage of the glandular epithelium, increased lymphocyte infiltration, and goblet cell loss. Noticeably, the TAN treatment improved the colon epithelial morphology, similar to that of the control group. MET treatment did not show any significant morphological improvement and restoration of the damaged structure of the glandular epithelium.

Evaluation of peripheral inflammation through expressions of plasma LPS and TNF-α levels was closely related to the integrity and permeability of the colon barrier. As shown in Figure 2D, the plasma LPS increased significantly in the model group in comparison with the control group (0.67 ± 0.14 vs*.* 0.43 ± 0.05 EU/mL, respectively, *p* = 0.0013). However, TAN intervention markedly reduced the elevated levels of LPS to 0.51 ± 0.07 EU/mL, which was significantly lower than that of the model group (*p* = 0.0316). The MET group did not show any significant change in LPS (0.61 ± 0.10 EU/mL) compared with the model group (*p* = 0.6574). The plasma LPS in the TAN group was not significantly different from that of the MET group (0.51 ± 0.07 vs*.* 0.61 ± 0.10 EU/mL, respectively, *p* = 0.2663).

Similarly, the plasma TNF-α was elevated significantly in the model group compared with the control group (748.32 ± 193.69 vs*.* 452.80 ± 65.33 pg/mL, respectively, *p* = 0.0012). TAN significantly reduced the plasma TNF-α level compared with the model group (545.00 ± 68.98 vs*.* 748.32 ± 193.69 pg/ml, respectively, *p* = 0.0251). The MET group showed a reduced level of TNF-α compared with the model group (644.81 ± 84.52 vs*.* 748.32 ± 193.69 pg/mL, respectively), though the change did not reach statistical significance (*p* = 0.3974). In addition, there was no significant difference in TNF-α between the TAN group and the MET group (*p* = 0.4280).

### 3.4 TAN Modulated the Neurological and Colon Tight Junction Biomarkers

**FIGURE 3:**
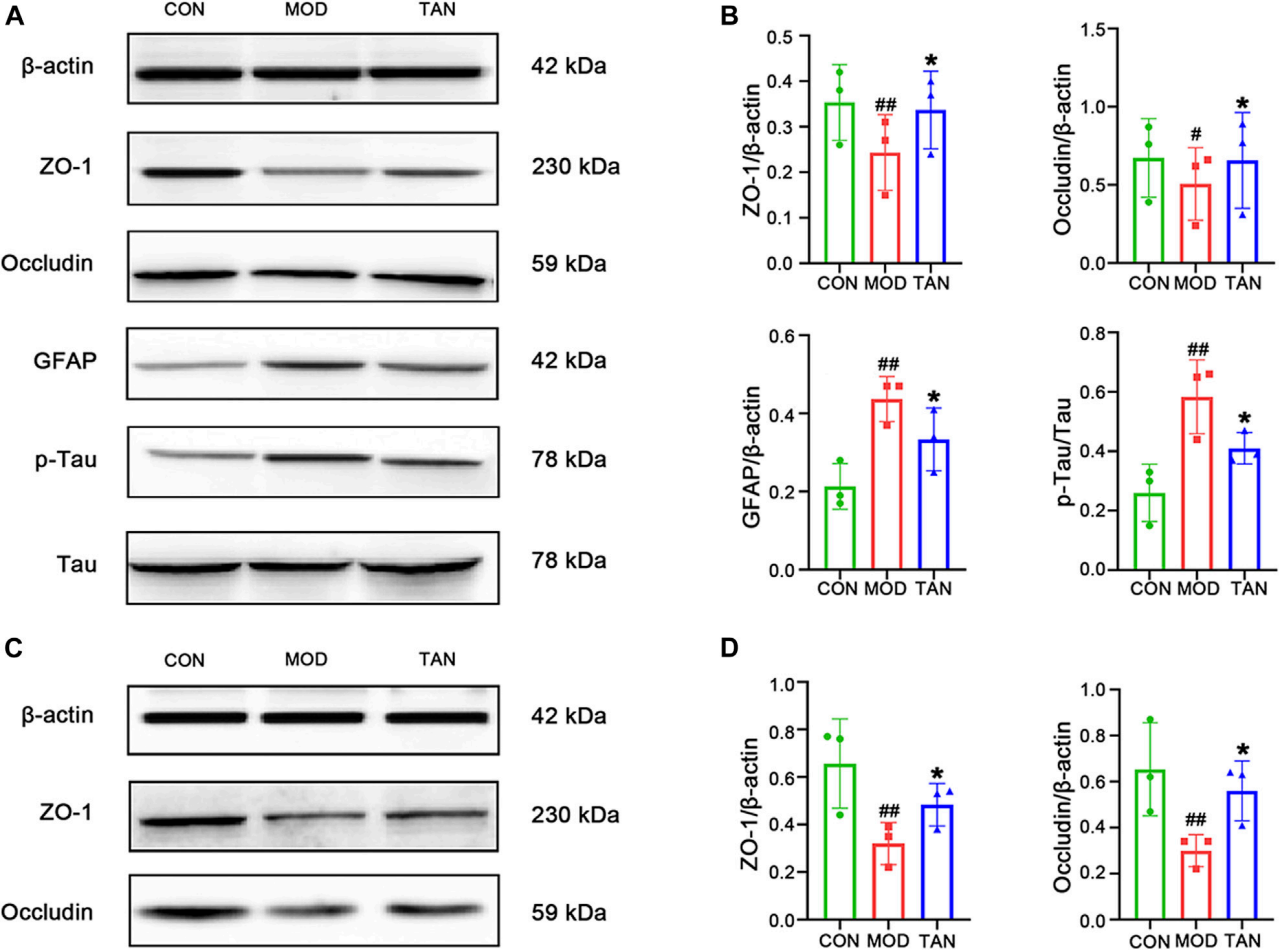
TAN restored the neurological and colon tight-junction biomarkers. **(A)** Representative Western blot images and **(B)** their statistical analysis of protein expressions of ZO-1, occludin, GFAP, p-Tau/Tau in the hippocampus (*n* = 3 independent experiments). ^#^ *p* < 0.05, ^##^ *p* < 0.01 vs*.* CON group and *p < 0.05 vs*.* MOD group by one-way ANOVA. **(C)**. Representative Western blot images from the three individual experiments and **(D)** their statistical analysis of protein expressions of ZO-1 and occludin in colon tissues (*n* ≥ 3). ^#^ *p* < 0.05 vs*.* CON group and *p < 0.05 vs*.* MOD group by one-way ANOVA. TAN, tanshinone IIA; CON, control; MOD, model; ANOVA, analysis of variance.

The brain tight-junction proteins, ZO-1 and occludin, were measured to evaluate the integrity of the blood–brain barrier (BBB). Since MET did not show a significant effect in improving spatial and memory function in the MWM test and in alleviating the pathological changes in the hippocampal and colon barrier, it was excluded from the following mechanistic studies. As shown in Figures 3A,B, ZO-1 and occludin expressions were significantly reduced in rats with HFD- and STZ-induced diabetes when compared with the control group (*p* = 0.0077 and *p* = 0.0189, respectively), suggesting an impaired tight-junction function of BBB in the model group. However, the TAN treatment revealed significant increases in ZO-1 (*p* = 0.0139) and occludin (*p* = 0.0270) protein expressions compared with the model group, suggesting that the TAN treatment was associated with a BBB protective effect. In addition, a significantly higher GFAP level was detected in the model group compared with the control group (*p* = 0.0016), suggesting increased astroglia activation after the HFD and STZ stimulation. The TAN treatment significantly reduced the GFAP level (*p* = 0.027) compared with the model group. Moreover, the TAN treatment significantly lowered the elevated p-Tau/Tau level when compared with the model group (*p* = 0.0303), highlighting the potential of TAN in ameliorating HFD- and STZ-induced Tau pathology. The Western blot images for each biomarker produced by the three individual experiments are shown in the Supplementary Material S1. Taken together, these results suggest that the TAN treatment attenuated the hyperglycaemia -induced hippocampal BBB impairment and neuron deficit.

The tight-junction proteins, ZO-1 and occludin, were measured to evaluate the integrity of the colon epithelial barrier. The protein expressions and statistical analysis of ZO-1 and occludin are shown in Figures 3C,D, respectively. Significant reductions in ZO-1 and occludin protein expressions were observed in the model group (*p* = 0.0038 and *p* = 0.0148, respectively). The TAN treatment restored the protein expressions of ZO-1 (*p* = 0.0462) and occludin (*p* = 0.0415) compared with the model group, suggesting that TAN attenuated HFD- and STZ-induced intestinal barrier impairment. The Western blot images for these two biomarkers obtained by the three individual experiments are shown in Supplementary Material S2.

### 3.5 TAN Regulated the Taxonomy of the Gut Metagenome in Diabetic Rats

#### 3.5.1 Diversity

**FIGURE 4:**
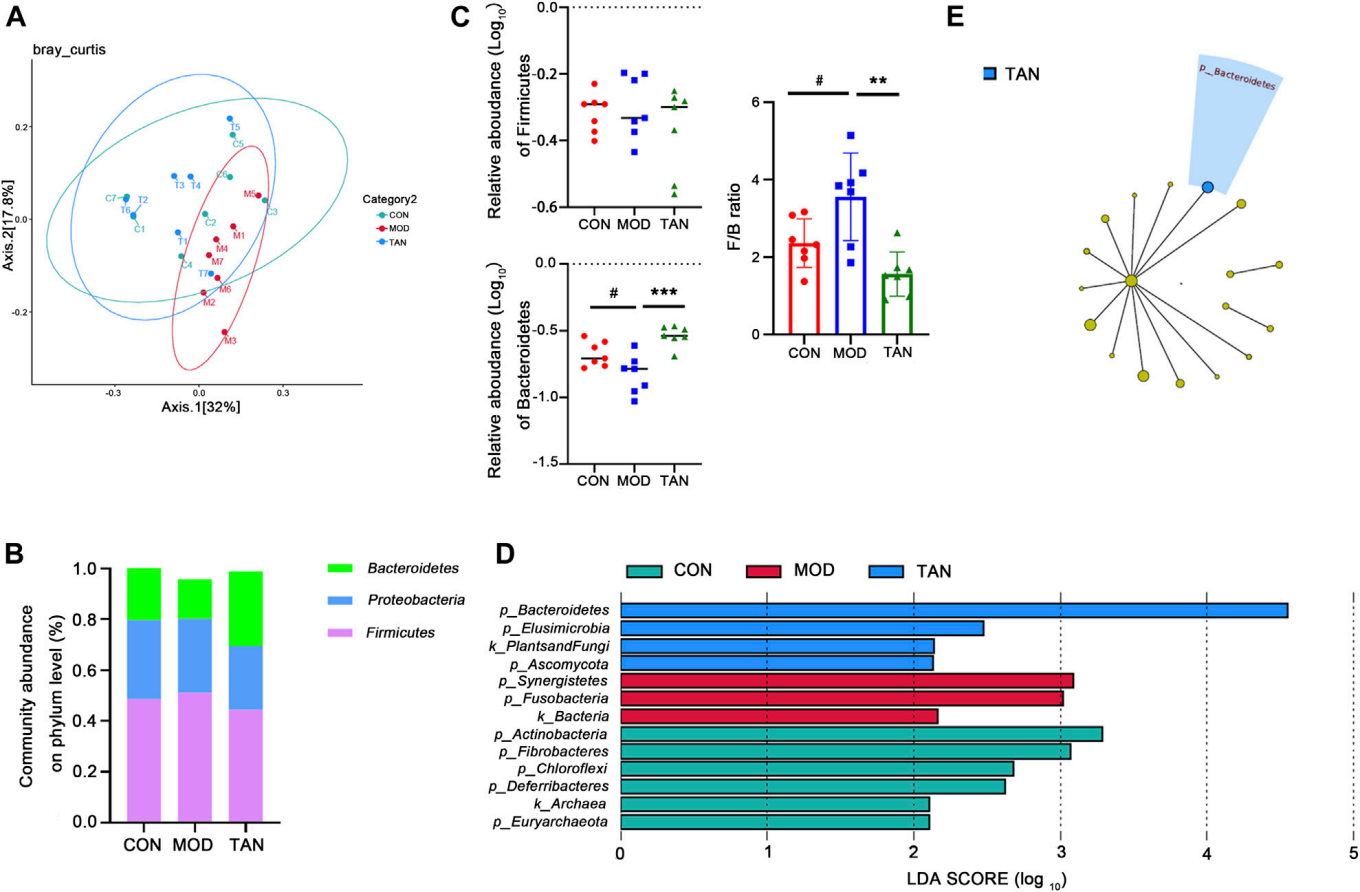
TAN restored the impaired diversity of the gut microbiome and regulated the gut microbiome at the phylum level (*n* = 7 rats in each group). **(A)** The β-diversity analysed by PCoA suggested that the distribution of the dots in the MOD group was distinguished from that in the CON group, whereas most of the samples in the TAN group were distributed similarly as in the CON group. **(B)** The relative abundance of the three top-ranked phyla among the three groups. **(C)** The relative abundance (log10) of *Firmicutes* and *Bacteroidetes* and their ratio compared among the three groups. The difference between the groups was assessed by one-way analysis of variance. ^#^ *p* < 0.05 vs*.* CON group and **p < 0.01, ***p < 0.001 vs*.* MOD group. **(D)** Feature phyla in the CON, MOD, and TAN groups at the LDA2 level as analysed by LEfSe. **(E)** Feature phyla in the TAN group at the LDA4 level as analysed by LEfSe. TAN, tanshinone IIA; CON, control; MOD, model; LDA, linear discriminant analysis; LEfSe, LDA effect size.

Metagenomic shotgun sequencing of seven fecal samples collected from each group was performed. Since MET did not show a significant effect in improving cognitive function, it was excluded from the following study for the modulation of the microbiome in relation to improved cognitive function. The β-diversity analysed by PCA, as shown in Figure 4A, suggests that the distribution of the seven samples in the model group was distinguished from that in the control group (principal component 1%: 17.8%, principal component 2%: 32%). By contrast, most of the samples in the TAN group were distributed in a domain similar to that in the control group, indicating that the TAN group shared a similar dominant species with the control group different from that of the model group.

#### 3.5.2 Abundance of Gut Metagenome at the Phylum Level

The gut microbial taxa that were influenced by TAN in the rats with HFD- and STZ-induced diabetes include a total of 16 phyla, 183 genera, and 399 species. At the phylum level, the top three dominant phyla were *Firmicutes*, Proteobacteria, and *Bacteroidetes*, which accounted for over 95% of the total abundance in the three groups (Figure 4B). Although no significant change was detected in the abundance of *Firmicutes* among the three groups (*p* = 0.4187), as shown in Figure 4C, the relative abundance of *Bacteroidetes* decreased significantly in the model group (*p* = 0.0220), and it was markedly restored in the TAN group (*p* = 0.0002 vs*.* the model group). Noticeably, the *Firmicutes*/*Bacteroidetes* ratio was significantly increased in the model group compared with the control group (*p* = 0.0472), whereas TAN markedly reduced it (*p* = 0.0019). In the LEfSe analysis (Figure 4D), three phyla were suggested as feature phyla in the TAN group at the LDA2 level: *Bacteroidetes* (LDA score = 4.549, *p* = 0.002), *Elusimicrobiota* (LDA score = 2.473, *p* = 0.042), and *Ascomycota* (LDA score = 2.133, *p* = 0.037); these were distinct from those in the model and control groups. At the LDA4 level, *Bacteroidetes* was picked up as the only predominant phylum in the TAN group, suggesting its high expression in this group (Figure 4E).

#### 3.5.3 Abundance at the Genus Level

**FIGURE 5:**
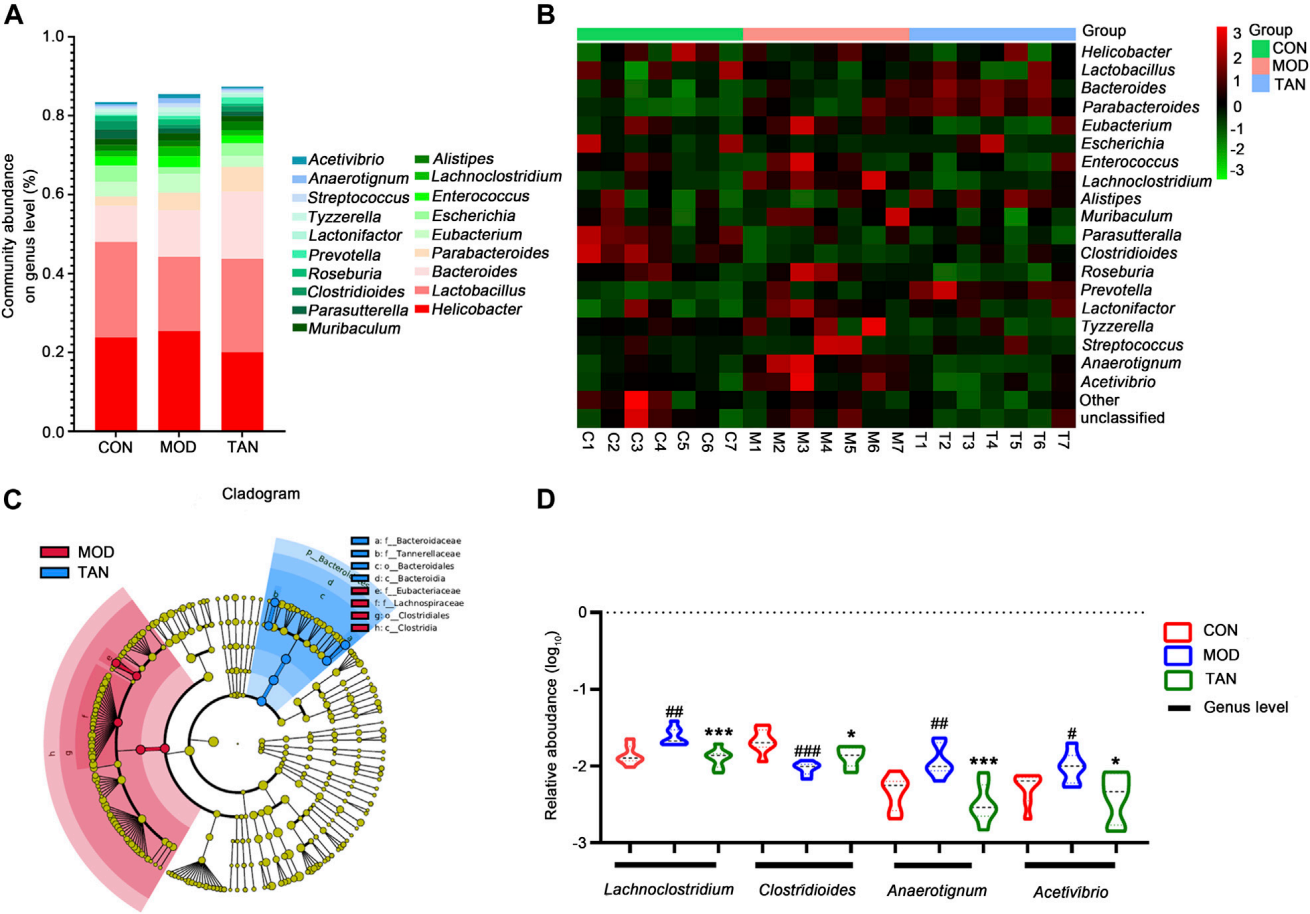
The effect of TAN on the abundance of the gut microbiome at the genus level (*n* = 7 rats in each group). **(A)** The relative abundance of the top-ranked genera among the three groups. **(B)** Hierarchical clustering heat map of the top differentiated taxa at the genus level among the three groups. **(C)** Feature genera in the MOD and TAN groups at the LDA4 level as analysed by LDA effect size. There were no feature genera detected in the CON group at the LDA4 level. **(D)** The comparison of the relative abundance (log10) of *Lachnoclostridium*, *Clostridioides*, *Anaerotignum*, and *Acetivibrio* in the three groups. ^#^ *p* < 0.05, ^##^ *p* < 0.01, ^###^ *p* < 0.001 vs*.* CON group and *p < 0.05, ***p < 0.001 vs*.* MOD group. Differences were examined by unpaired *t* test. TAN, tanshinone IIA; CON, control; MOD, model; LDA, linear discriminant analysis.

At the genus level, the top 10 dominant genera in the three groups were identified as *Helicobacter*, *Lactobacillus*, *Bacteroides*, *Parabacteroides*, *Eubacterium*, *Escherichia*, *Enterococcus*, *Lachnoclostridium*, *Alistipes*, and *Muribaculum* constituting over 80% of the overall detected abundance of the genus in all these groups (Figure 5A). The heat map in Figure 5B shows the clustering of the top 19 differentiated taxa. The microbiome community composition in most of the TAN-treated animals seemed to be comparable with that of the control group, whereas a larger variety was shown in the model group. LEfSe analysis (Figure 5C) suggested that the feature genera in the TAN group with high expressions were *Bacteroidaceae* (LDA score = 4.592, *p* = 0.007), Tannerellaceae (LDA score = 4.296, *p* = 0.002), *Bacteroidales* (LDA score = 4.844, *p* = 0.002), and *Bacteroidia* (LDA score = 4.844, *p* = 0.002), which were distinct from those in the model group. These four feature genera all belong to the *Bacteroides* phylum, which was dominant in the TAN-treated group. Compared with the control group, the relative abundance (log10) of *Lachnoclostridium* (*p* = 0.0023), *Anaerotignum* (*p* = 0.0042), and *Acetivibrio* (*p* = 0.0263) were significantly increased, whereas *Clostridioides* was decreased (*p* = 0.0002) in the model group. The TAN treatment markedly reduced the levels of *Lachnoclostridium* (*p* = 0.0009), *Anaerotignum* (*p* = 0.0007), and *Acetivibrio* (*p* = 0.0151) and restored the level of *Clostridioides* (*p* = 0.0213) in comparison to the model group (Figure 5D), suggesting its regulatory ability for those genera.

#### 3.5.4 Abundance at the Species Level

**FIGURE 6:**
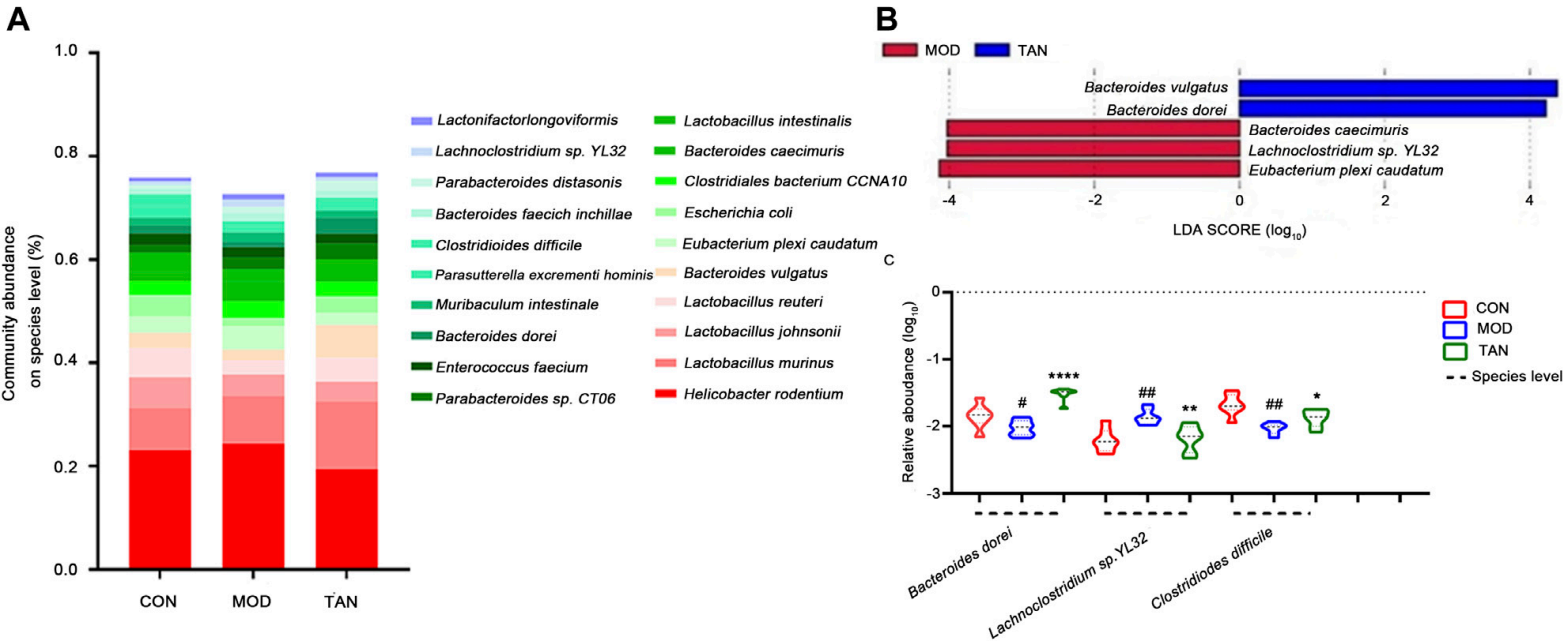
The effect of TAN on the abundance of the gut microbiome at the species level (*n* = 7 rats in each group). **(A)** The relative abundance of the top-ranked species among the three groups. **(B)** Feature species in the MOD and TAN groups at the LDA4 level as analysed by LEfSe. There was no feature species detected in the CON group at the LDA4 level. **(C)** The comparison of the relative abundance (log10) of *Bacteroidetes dorei*, *Lachnoclostridium* sp. YL32, and *Clostridiodes difficile* in the three groups. ^#^ *p* < 0.05, ^##^ *p* < 0.01 vs*.* CON group and *p < 0.05, **p < 0.01, ****p < 0.0001 vs*.* MOD group. Differences were examined by unpaired *t* test. TAN, tanshinone IIA; CON, control; MOD, model; LDA, linear discriminant analysis.

The changed abundance of the gut microbiome was further specified at the species level. The top five dominant species identified in the three groups were ranked from *Helicobacter rodentium*, *L. murinus*, *L. johnsonii*, and *L. reuteri* to *Bacteroides vulgatus* (Figure 6A). LEfSe analysis (Figure 6B) at the LDA4 level suggested that the feature species in the TAN group included *B. dorei* (LDA score = 4.011, *p* = 0.001) and *B. vulgatus* (LDA score = 4.311, *p* = 0.002), which were both from the *Bacteroides* phylum, as opposed to the feature species in the model group. Group comparison for relative abundance (log10) confirmed that *B. dorei* was significantly restored in the TAN group (*p* < 0.0001) as compared with the model group. In addition, TAN significantly reduced the excessive expressions of *Lachnoclostridium* sp. YL32 (*p* = 0.0027) and *C. difficile* (*p* = 0.0213) which corresponded to the change in *Lachnoclostridium* and *Clostridioides* at the genus level (Figure 6C).

#### 3.5.5 TAN Regulated the Functional Components of Metagenome in Diabetic Rats

**FIGURE 7:**
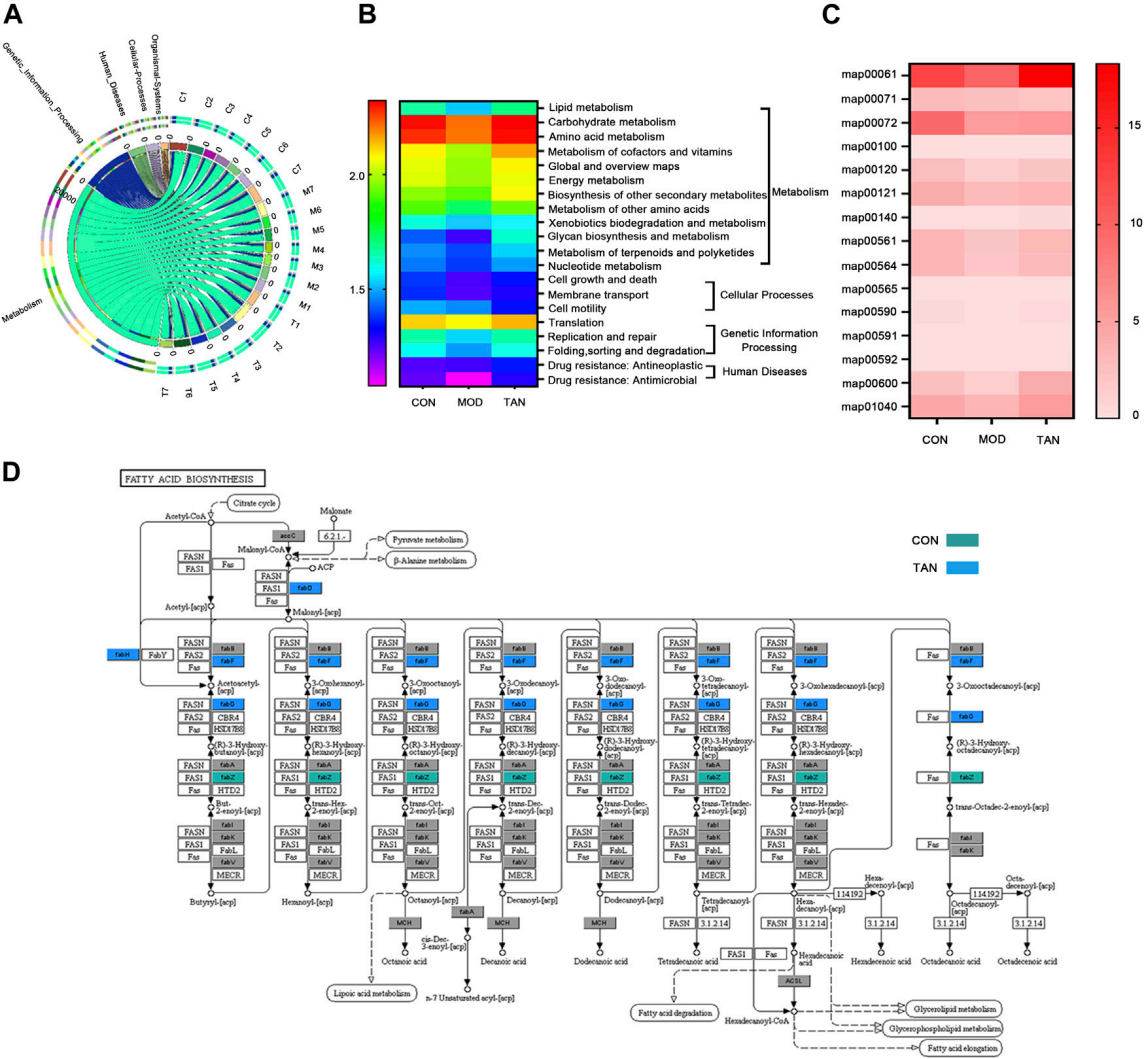
Functional components in the gut microbiome that were modulated by TAN against HFD and STZ-induced diabetes using the HUMAnN2 program (*n* = 7 rats in each group). **(A)** Altered KEGG pathways (level 1) among the three groups as shown in the Circos plots. **(B)** Heat map of the altered KEGG pathways (level 2) among the three groups. **(C)** Heat map of the altered KEGG mapping among the three groups. **(D)** KEGG map00061 with altered genes in the CON and TAN groups. TAN, tanshinone IIA; KEGG, Kyoto Encyclopedia of Genes and Genomes.

In total, 462 out of 12,207 KOs were differentially abundant between the control and model groups (Dunn’s test: *p* < 0.05), and 609 were differentiated between the model and TAN groups. In particular, 23 altered KOs in the model group were significantly restored by the TAN treatment. This corresponded to five KEGG pathways (three at level 1 and two at level 2) and 11 KEGG modules that showed significant differences according to the *p* values in Dunn’s test. It was evident that the metabolism-related function was the most regulated functional component (level 1) by TAN, as shown in the Circos plots (Figure 7A). The detailed comparison of the changed KEGG pathways (level 2) among the three groups is illustrated in the heat map (Figure 7B), in which the pattern of the pathways’ enrichment is distinct between the control and model groups, whereas the TAN group generally shows a pattern of pathways consistent with that of the control group. In addition, 12 pathways were significantly different between the model and TAN groups (all *p* values < 0.05), as analysed by Dunn’s test, and 8 pathways were restored to a comparable level with that in the control group.

Specifically, the altered maps in the lipid metabolism–related pathways in the three groups were examined. As shown in Figure 7C, most of the maps were downregulated in the model group in comparison with the control group. In contrast, most of the maps were enriched by the TAN treatment, which then showed a comparable pattern with the control group. Particularly, TAN revealed the most evident regulatory effect on map 00061, which was on fatty acid biosynthesis. The detailed map 00061 is shown in Figure 7D, with *FabG* and *FabF* as the feature genes regulated by TAN. Upregulation of map 00061 activated the n-7 unsaturated acyl-[acp] and was linked to lipoic acid metabolism, fatty acid degradation, glycerolipid metabolism, glycerophospholipid metabolism, and fatty acid elongation.

#### 3.5.6 TAN Modulated Fecal Short-Chain Fatty Acid Profiling in Diabetic Rats

**FIGURE 8:**
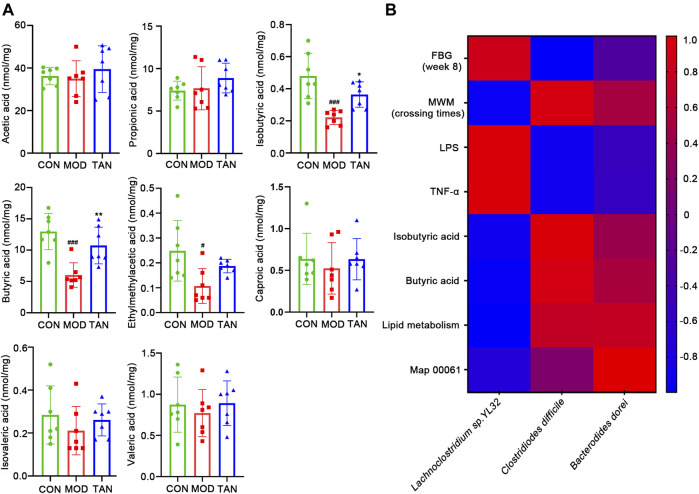
TAN restored the fecal SCFAs in diabetic rats, and the correlation between the changed species responding to TAN and the diabetes-related index (*n* = 7 rats in each group). **(A)** Comparison of identified SCFAs in the three groups analysed by one-way ANNOVA test. ^#^ *p* < 0.05, ^###^ *p* < 0.001 vs*.* CON group and *p < 0.05, **p < 0.01 vs*.* MOD group. **(B)** Correlation heat map of changed species responding to TAN and the diabetes-related index, including FBG, MWM (platform-crossing times), LPS, TNF-α, butyric acid, lipid metabolism, and map 00061. Red color represents positive correlation, and blue color represents negative correlation. TAN, tanshinone IIA; SCFA, short-chain fatty acid.

Since the lipid metabolism pathways have been shown as the leading metabolic pathways corresponding to the regulation of microbiota by TAN, examination of the key SCFA levels in the fecal samples as part of the lipid metabolism among the three groups was carried out. As shown in Figure 8A, HFD- and STZ-induced diabetes significantly reduced the amount of isobutyric acid (*p* = 0.0010), butyric acid (*p* = 0.0004), and 2-methylacetic acid (*p* = 0.0250). The TAN treatment significantly reversed the reduction in isobutyric acid (*p* = 0.0464) and butyric acid (*p* = 0.0076). The TAN treatment was also associated with restoring 2-methylacetic acid; however, the change did not reach statistical significance (*p* = 0.2344) by one-way ANOVA analysis.

The correlations between the key SCFAs and the biomarkers of DCI including FBG, MWM (platform-crossing times), LPS, TNF-α, isobutyric acid, butyric acid, and map 00061 were investigated. As shown in Figure 8B, the species that were upregulated by the TAN treatment, *C. difficile* and *B. dorei*, were both negatively correlated with FBG (*r* = −0.9913 and *r* = −0.2873, respectively), LPS (*r* = −0.9231 and *r* = −0.5262, respectively), and TNF-α (*r* = −0.9164 and *r* = −0.54606, respectively). *C. difficile* and *B. dorei* were positively correlated with MWM (*r* = 0.9309 and *r* = 0.5083, respectively), isobutyric acid (*r* = 0.9667 and *r* = 0.4059, respectively), butyric acid (*r* = 0.9199 and *r* = 0.5331, respectively), lipid metabolism (*r* = 0.7419 and *r* = 0.7797, respectively), and map 00061 (*r* = 0.1556 and *r* = 1.00, respectively), although the correlations did not reach statistical significance (*p* values >0.05). A species that was reduced by the TAN treatment, *Lachnoclostridium* sp. YL32, was positively correlated with FBG (*r* = 0.8650), LPS (*r* = 0.9565), and TNF-α (*r* = 0.9699). However, none of the correlations reached statistical significance (*p* values >0.05).

## 4 Discussion

In the current study, it was demonstrated that both TAN and MET produced strong anti-hyperglycaemic effects in rats with HFD- and STZ-induced diabetes. Furthermore, TAN exhibited a potent effect on spatial learning and memory function and regulated the levels of neurological biomarkers in these diabetic rats; these effects were generally not observed in the MET group. These results are consistent with the findings of previous studies that showed that TAN treatment ameliorated cognitive deficits and improved spatial learning in diabetic mice and that the mechanism was associated with attenuation of endoplasmic-reticulum stress-induced apoptosis (Chen et al., 2018; He et al., 2020; Xu et al., 2020) and antioxidant properties (Liu et al., 2010). The present study explored the possible effect of TAN on the regulation of the gut microbiome, which provided new insight into the diverse mechanism underlying TAN’s effect on DCI. For the first time, this study has shown that TAN corrects the abundance of *B. dorei*, *Lachnoclostridium* sp. YL32, and *C. difficile* and may play a key role in microbiome regulation and consequently contribute to restoring the levels of SCFAs, repairing the colon barrier, reducing systemic inflammation, and improving neurologic deficit.

Our results show that an 8-week treatment of TAN significantly reduced the FBG level, which is consistent with previous studies in rats with STZ-induced diabetes and amyloid precursor protein/presenilin 1 transgenic mice (Chen et al., 2018; He et al., 2020; Xu et al., 2020). TAN also alleviated the diabetes-related impairments in cognitive and memory function, the endothelial barrier, and neurological biomarkers. Although MET exhibited an excellent effect in reducing FBG levels, its ability to improve cognitive function was limited in this study. Previous studies have suggested that MET could significantly improve cognitive dysfunction in patients with type II diabetes (Zhang et al., 2020). These conflicting results may be due to the short administration regimen in the present study. Particularly, previous studies reported that long-term use of MET (100 mg/kg in drinking water daily for 6 months) in normal male Wistar rats or administration for more than 6 years in older persons was required for MET to exhibit a significant neuroprotective effect (Ng et al., 2014; Gorgich et al., 2021).

Furthermore, the underlying mechanism of TAN for improving cognitive function was explored in this study. Impaired morphology of the colon tight junction and reduced ZO-1 and occludin expressions were observed in the rats with HFD- and STZ-induced diabetes. The intestinal barrier function is critical for maintaining homeostasis and integrity of the gut, and damage to the barrier could expose the epithelial cells to bacteria/bacterial products, leading to the activation of immune cells *via* toll-like receptor 4 (TLR4)/myeloid differentiation primary response 88–dependent signalling pathways attributed to the gut bacteria–derived LPS, and the translocation of these to the systemic circulation (Ghosh et al., 2020). Indeed, the results from this study demonstrated higher circulating levels of LPS and TNF-α in the diabetic animals, implying impairment of the intestinal barrier function (Ghosh et al., 2020). Remarkably, the TAN treatment appeared to be able to repair the colon tight junction, restore the protein expressions of ZO-1 and occludin, and inhibit the circulating levels of LPS and TNF-α. These results led to further exploration of the underlying mechanistic action of TAN in regulating the microbiome and its products/metabolites.

Based on the microbiome profile post-trial in the control, model, and TAN groups, the results from this study showed that TAN exhibited a noticeable regulatory effect on the overall diversity and abundance of the dominant taxa of the gut microbiome. At the phylum level, changes in *Firmicutes* and *Bacteroidetes* among the three groups were specifically measured, which constitute over 50% of the total abundance of the phyla. It is generally agreed that the abnormal increase in the ratio of *Firmicutes*/*Bacteroidetes* is linked to obesity (Farràs et al., 2020), which was observed in the model group of this study. The results from this study showed that TAN significantly reduced the *Firmicutes*/*Bacteroidetes* ratio. It is worth noting that TAN significantly restored the level of *Bacteroidetes* but the effect on *Firmicutes* was minimal. Following this, alterations in genera and species that were associated with the regulatory activity of TAN in the microbiome were further explored. At the genera level, TAN was associated with high abundance of *Bacteroidales* and *Bacteroidia*, both of which belong to the *Bacteroidetes* phylum, highlighting the importance of this phylum in the TAN-induced effects.

The shotgun metagenomics has enabled the identification of the altered microbiome at the species level in this study; which was comparatively more informative than the 16S rRNA gene analysis. The latter can only detect the microbiome changes at the genus level (Peterson et al., 2021). In the present study, the key species that were changed by TAN were *B. dorei*, *Lachnoclostridium* sp. YL32, and *C. difficile*. The species of *Lachnoclostridium* sp. YL32 and *C. difficile* corresponded to the altered genera of *Lachnoclostriudium* and *Clostridiodes* observed in the tested animals, and the restored *B. dorei* species corresponded to the increased level of *Bacteroidetes* with TAN observed at the phylum level. The functional role of *Lachnoclostridium* sp. YL32 is unclear. A metagenomic analysis by Liang et al. (2020) showed that *Lachnoclostridium* sp. YL32 was significantly increased in adenoma and to a lesser extent in colorectal cancer in human fecal samples (Liang et al., 2020). In this study, a significant increase in the abundance of *Lachnoclostridium* sp. YL32 was observed in the rats with HFD- and STZ-induced diabetes, whereas the TAN treatment significantly reduced its expression. Further studies are warranted to explore the underlying mechanism of TAN treatment on this species in diabetes and DCI.

*C. difficile*, a Gram-positive bacteria, is suggested to cause pseudomembranous colitis, swelling or inflammation of the colon (Nomlomo and Nana, 2020). A retrospective study of the Nationwide Readmissions Database in the United States suggested that diabetes mellitus correlates to 30-day all-cause readmissions for *C. difficile*–induced enterocolitis (Kichloo et al., 2022). In addition, Lee et al. (2021) investigated the impact of diabetes mellitus on the treatment of *Clostridium butryricum* for mild to moderate *C. difficile* infection, and their results revealed that diabetes mellitus in affected individuals is a risk factor for treatment failure (Lee et al., 2021), suggesting the possible detrimental role of diabetes mellitus in *C. difficile* infection. Interestingly, this study has demonstrated for the first time that TAN restored the decreased abundance of *C. difficile* in the group with HDF- and STZ-induced diabetes. The role of this species associated with the pathogenesis of diabetes mellitus and the regulatory activity of TAN is worth future investigation.

Previous studies have suggested that *B. dorei* is associated with the pathogenesis of type I diabetes. A human cohort study demonstrated a strong link between *B. dorei* and the disease in Finnish children at high risk for type I diabetes (Davis-Richardson et al., 2014). Moreover, it was revealed that the LPS released from *B. dorei* antagonized the *Escherichia coli* LPS, leading to the inhibition of TLR4-mediated immune stimulation and inflammatory cytokine responses (Vatanen et al., 2016; Knip and Honkanen, 2017). In the present study, TAN significantly restored the decreased abundance of *B. dorei* in the rats with HFD- and STZ-induced diabetes. This may lead to a reduction in systemic information and neuroprotective effect. Further investigation is warranted to explore this mechanistic pathway.

The functional analysis in this study showed that lipid metabolism played an important role in the regulatory effect of TAN on the gut microbiome. In particular, map 00061 was evidently enriched by the TAN treatment, which relates to fatty acid biosynthesis *via* the modulation of *fabF* and *fabG*. It was demonstrated that the FabF homologue located within the fatty acid biosynthetic gene cluster of *Clostridium acetobutylicium* functions in the synthesis of both unsaturated and saturated fatty acids (Zhu et al., 2009). Following this, several key SCFAs were explored to examine the specific regulatory activities of TAN. It was identified that TAN significantly restored the reduced production of isobutyric acid and butyric acid. Both SCFAs are known to affect the pathogenesis of Alzheimer’s disease through the inhibition of microglia- and astrocyte-induced inflammation, and aggregations of Aβ and Tau (Kumar et al., 2010; Macfarlane and Macfarlane, 2012). The butyric acid supplementation was shown to markedly increase the activities of serum and renal antioxidant enzymes, including superoxide dismutase, catalase, and glutathione peroxidase, and inhibit lipid peroxidation in rats with STZ-induced diabetes (Kumar et al., 2010). Another study suggested that butyric acid treatment at 500 mg/kg body weight/day was effective in controlling the diabetic status of these rats (Kumar et al., 2002). Moreover, butyric acid was also found to effectively reduce inflammation and was critical in maintaining the healthy function of the intestinal barrier (Stachowska et al., 2021). Thus, the TAN-induced increase in butyric acid production may contribute to improvement of lipid metabolism and circulatory inflammation, leading to protection of cognitive function against diabetes. To confirm the link between SCFAs (isobutyric acid and butyric acid), cognitive function–related factors (FBG, MWM test, and systemic inflammation), and TAN-induced genera changes, a Pearson correlation analysis was conducted among these environmental factors and the three key species. In particular, the results revealed that butyric acid may be involved in the negative regulation of *Lachnoclostridium* sp. YL32 and positive regulation of *C. difficile* and *B. dorei*. The latter two species were both positively correlated with MWM (platform-crossing times) but negatively with FBG, LPS, and TNF-α. This finding suggested the positive role of *C. difficile* and *B. dorei* as well as their metabolites of isobutyric acid and butyric acid in the regulation of diabetic and DCI. In contrast, *Lachnoclostridium* sp. YL32 played a negative role in attenuating FBG level, inflammation, and cognitive dysfunction. Further studies are warranted to confirm the specific genera that produce SCFA metabolites contributing to improved DCI. In addition, it is worth mentioning that the inclusion of microbiome analysis pre-treatment as a baseline may strengthen the research findings from the present study.

## 5 Conclusion

An 8-week oral administration of TAN showed a marked reduction of FBG in the rats with HFD- and STZ-induced diabetes. TAN significantly improved cognitive and memory function, as assessed by MWM tests. TAN repaired the impaired intestinal barrier integrity and reduced the circulated pro-inflammatory mediators, including TNF-α and LPS, which was likely to be associated with gut microbiota regulation. TAN improved the overall diversity and corrected the relative abundance of the *Firmicutes/Bacteroidetes* ratio at the phylum level. The species altered by TAN were identified as *Lachnoclostridium* sp*.* YL32, *C. difficile*, and *B. dorei*. The functional components altered by TAN were largely associated with lipid metabolism and biosynthesis of fatty acids. Specifically, it was identified that TAN restored the production of isobutyric acid and butyric acid, which were positively linked to *C. difficile* and *B. dorei* and negatively corresponded to *Lachnoclostridium* sp. YL32. Taken together, these findings provide new evidence to support the use of TAN as a promising phytochemical for the management of DCI. Moreover, this study revealed that the mechanisms underlying the neuroprotective effect of TAN were likely to be associated with the regulation of the gut microbiome and metabolic SCFAs through the gut–brain axis. New evidence on the modulation of certain bacterial species by TAN may shed light on novel therapeutic agents for tackling DCI through a similar approach.

## Data Availability

The datasets presented in this study can be found in online repositories. The names of the repository/repositories and accession number(s) can be found at https://www.ncbi.nlm.nih.gov/bioproject/818720.
