# Supplementary material for: Effect of Tanshinone IIA on Gut Microbiome in Diabetes-Induced Cognitive Impairment
Source: Front Pharmacol. 2022 Jul 11;13:890444. doi: 10.3389/fphar.2022.890444 (PMC9309808; doi:10.3389/fphar.2022.890444)
Supplement: Supplementary file 1 [file Table1.DOCX]

Supplementary Material

## Supplementary

##
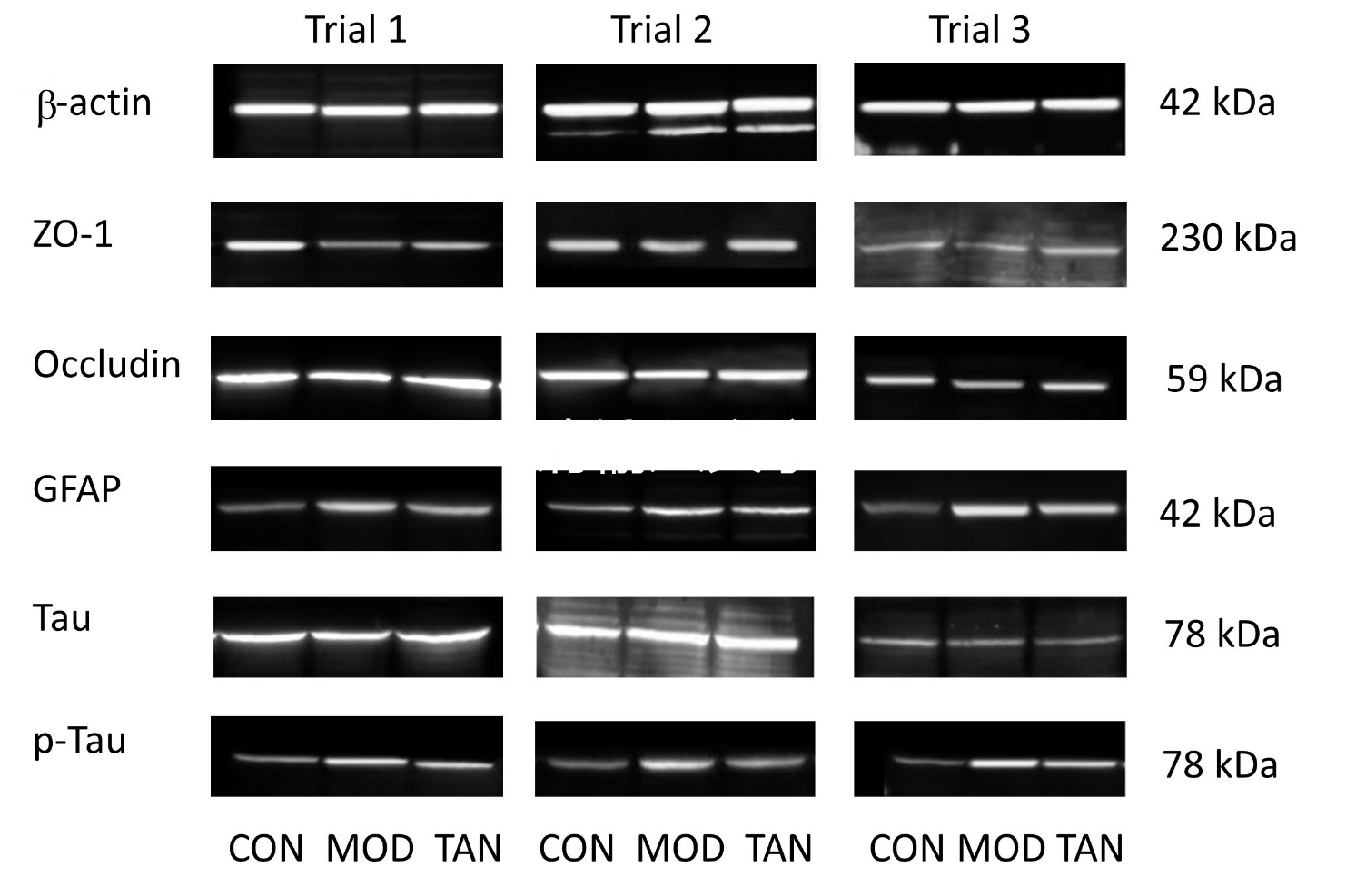
 Supplementary Figure 1. Western blot images of ZO-1, occludin, GFAP, p-tau, tau and β-actin in hippocampus tissues collected from each group (n=3 experiments), CON: Control group; MOD: Model group; TAN: TAN treatment group; MET: metformin treated group.


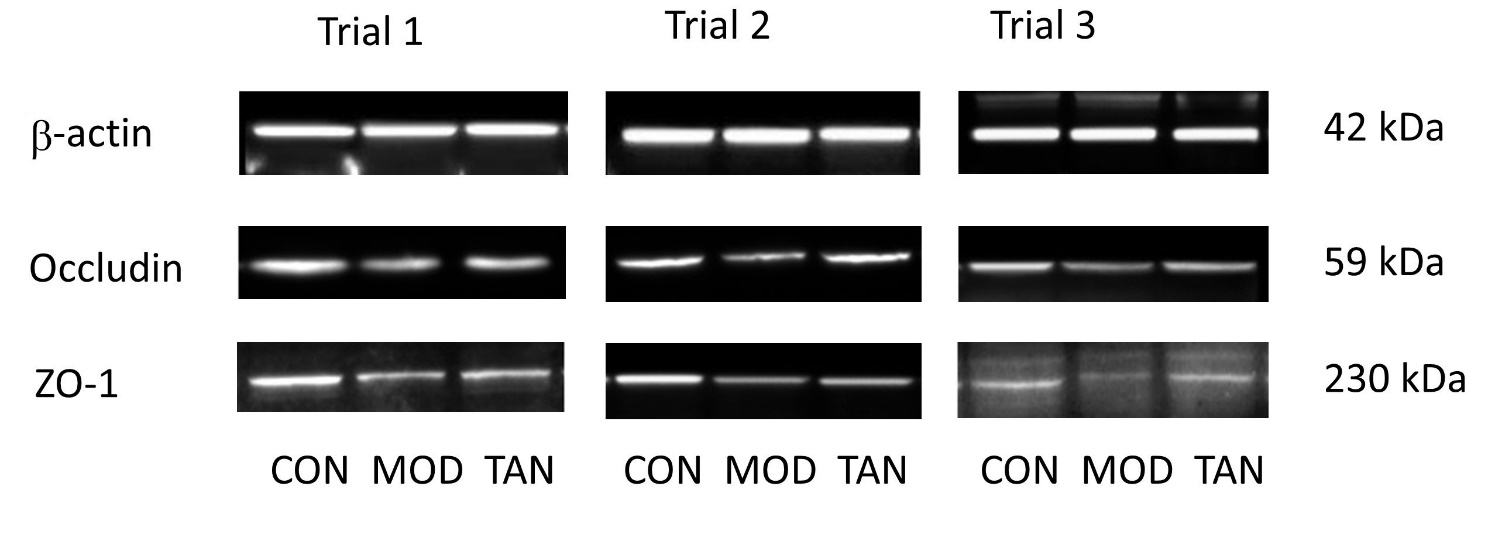


**Supplementary Figure 2.** Western blot images of occludin, ZO-1 and β-actin in colon tissues collected from each group (n=3 independent experiments), CON: Control group; MOD: Model group; TAN: TAN treatment group; MET: metformin treated group.
